# *Lactobacillus plantarum* synergistically regulates M1 macrophage polarization in resistance against *Salmonella enterica* serovar Typhimurium infection

**DOI:** 10.3389/fmicb.2022.933550

**Published:** 2022-10-06

**Authors:** Bingjie Duan, Ruihan Liu, Yumeng Shi, Anqi Sun, Zhengxu Tang, Chunfeng Wang, Jingtao Hu

**Affiliations:** ^1^College of Animal Science and Technology, Jilin Agricultural University, Changchun, China; ^2^Jilin Provincial Key Laboratory of Animal Microecology and Health Breeding, Changchun, China; ^3^Key Laboratory of Animal Production and Product Quality Safety of Ministry of Education, Changchun, China

**Keywords:** *Lactobacillus plantarum*, *S*. Typhimurium, macrophage polarization, BMMs, M1

## Abstract

Macrophage polarization affects the progression of pathogenic bacterial infections. *Lactobacillus* is widely used to interact with macrophages and to exert specific immunomodulatory activities. In this study, we investigated the regulation of macrophage polarization against *Salmonella enterica* serotype Typhimurium (STM) by *Lactobacillus plantarum* JL01 (LP), to explore prevention and treatment strategies for salmonellosis. We assessed the *in vitro* differential polarization of RAW 264.7 macrophages and mouse bone marrow macrophages (BMMs) by LP against STM, by measuring protein and cytokine levels, and bactericidal activity. In addition, we assessed the protective effects of LP against STM by evaluating weight loss, survival, the burden of STM in tissues, the polarization of macrophages in the spleen and mesenteric lymph nodes (MLNs), intestinal histopathology, and cytokine production. LP slightly affected the polarization of RAW 264.7, a slight M1-skewing. LP promoted the RAW 264.7 bactericidal activity against STM. In BMMs, M1 polarization induced by LP was significantly lower than the M1-positive phenotype. The combination of LP with M1 synergistically improved M1 polarization and bactericidal activity against STM compared to the individual effects. LP promoted the activation of the NF-κB signaling pathway. Supplementation with the NF-κB inhibitor decreased M1 polarization induced by LP. We observed the protective effect of LP against STM in C57BL/6 mice, through a decrease in weight loss, mortality, STM burden in the liver, and promotion of macrophage M1 and M2 polarization in the spleen and MLNs; though M1 was higher, it did not cause inflammatory damage. In summary, LP can synergistically promote M1 polarization in combination with the M1 phenotype through the NF-κB signaling pathway and increases resistance against *S*. Typhimurium infection. These findings will lay the foundation for the prevention and treatment of *S*. Typhimurium infections in the future.

## Introduction

*Salmonella enterica* serotype Typhimurium (*S*. Typhimurium, STM) is a typical *Salmonella* serotype. Foodborne pathogens are mostly associated with gastroenteritis, which is characterized by diarrhea, fever, and intestinal inflammation (Lathrop et al., 2018). STM has evolved multiple mechanisms to evade host immune defenses and ensure persistent transmission (Boyle et al., 2006); its ability to survive and replicate in macrophages is one such crucial strategy (Monack, 2012). STM can disrupt the endosome trafficking pathway to form *Salmonella*-containing vesicles (SCVs), which contribute to the survival of intracellular *Salmonella* (Cheminay et al., 2005). In addition, *Salmonella* infection leads to pyroptosis of macrophages; this leads to their release from the cytosol of nutrient-rich host cells into the extracellular environment, promoting intestinal inflammation and further systemic infection (Jones et al., 2008). In addition, specific *Salmonella* strains can alter macrophage polarization, thereby promoting survival (Thurston et al., 2016; Amano, 2019).

Macrophages are important innate immune cells of the host that are widely distributed in the intestinal mucosa (Gogoi et al., 2019; Locati et al., 2020). Macrophages can be induced to differentiate into M1 (classical activation) or M2 (alternative activation) phenotypes by different environmental stimuli, such as pathogenic infection, cytokines, and tumors (Mantovani et al., 2002; Benoit et al., 2008; Atri et al., 2018; Thapa and Lee, 2019). M1 macrophage polarization is triggered by IFN-γ and LPS (Christoffersen et al., 2014). M1 macrophages can produce iNOS, express co-stimulating molecules (CD86^+^ and CD80^+^), and secrete pro-inflammatory cytokines, such as TNF-α, IL-1β, and IL-6. These properties of M1 macrophages are associated with resistance against pathogenic invasion (Wang et al., 2019). In contrast, the M2 phenotype can be polarized by IL-4 or IL-13. M2 macrophages suppress inflammatory injury and promote wound healing through Arg-1, high expression of CD206, and secretion of IL-10 (Viola et al., 2019; Zhen et al., 2021). Therefore, M1 macrophage polarization is assessed by evaluating the levels of pro-inflammatory cytokines or co-stimulatory molecules (CD80^+^ or CD86^+^), or iNOS. The anti-inflammatory cytokines CD206 or Arg-1 can be used to identify the M2 phenotype.

Macrophages are a part of the innate immune response; they recognize *Salmonella*-derived pathogen-associated molecular patterns (PAMP) through pattern recognition receptors (PRRs), such as Toll-like receptors (TLR) and NOD-like receptors (NLR) (Roszer, 2015). Macrophage polarization may play a decisive role in *Salmonella* infection (Gogoi et al., 2019). Chronic infection with *Salmonella* frequently results in polarization of M2 macrophages, which may lead to bacterial survival and replication (Fields et al., 1986). Therefore, altering M1 polarization may be an effective strategy for eliminating and killing *Salmonella*.

*Lactobacillus* is widely studied for the prevention and treatment of pathogenic infections (Biswas and Mantovani, 2012). Its beneficial effects have been accepted for decades; however, the exact mechanism remains unclear; its interaction with macrophages or dendritic cells could be a key factor (Sekirov et al., 2010; Stecher and Hardt, 2011). *Lactobacillus* promotes macrophage polarization by mediating cytokine secretion *via* the JAK-STAT and NF-κB signaling pathways (Manichanh et al., 2012; Eisele et al., 2013). Increasing evidence suggests that the NF-κB signaling pathway is involved in M1 macrophage polarization (Jiao et al., 2021). However, specific *Lactobacillus* strains have different effects on macrophage polarization, depending on the bacterial strain and the macrophage stimulator in that context (Habil et al., 2011). The lactic acid bacteria strain (EJ-1) did not promote TNF-α production when interacting with naïve macrophages, which significantly reduced the interaction of TNF-α with LPS-stimulated macrophages (Jang and Min, 2020). The probiotic, *Lactobacillus rhamnosus* GG (LGG) exhibited the immunotherapeutic potential to modify the host defense against pathogen invasion by promoting M1 polarization in bone marrow macrophages (BMMs) (Rocha-Ramírez et al., 2017). Therefore, it is necessary to elucidate the exact benefits and mechanisms of action of specific *Lactobacillus* strains.

In this study, we investigated the effect of *Lactobacillus plantarum* on the polarization of different macrophages to modulate resistance against STM infection *in vivo*. We also evaluated its protective effects against STM in C57BL/6 mice.

## Materials and methods

### Ethics statement

Sixty SPF female C57BL/6 mice (7 weeks old) were purchased from the Yisi Laboratory Animal Technology Co., Ltd. (Changchun, China). All procedures were performed according to the guidelines and standards approved by the Animal Welfare and Research Ethics Committee of the Jilin Agricultural University (approval number: JLAU 20200704001).

### Strains and growth

*Lactobacillus plantarum* JL01 (LP) was isolated from the intestinal tract of piglets in our previous study (CGMCC No.18056). LP and *Salmonella enterica* serovar Typhimurium (ATCC14028) were stored in the Jilin Provincial Engineering Research Center of Animal Probiotics. LP was cultured anaerobically in the Man-Rogosa-Sharpe (MRS) broth (Solarbio, China) at 37°C for 24 h, and STM was cultured in the Luria-Bertani (LB) broth (Solarbio, China) at 37°C for 12 h.

### Cell culture and lineage

Cells from the mouse monocyte-macrophage RAW 264.7 cell line (ATCC NO. TIB-71), maintained at the Jilin Provincial Engineering Research Center of Animal Probiotics, were cultured in RPMI-1640 (Gibco, USA) supplemented with 10% fetal bovine serum (FBS; Gibco, USA) in a humidified incubator at 37°C with 5% CO_2_.

### Bacterial co-culture with raw 264.7

Bacterial co-culture of RAW 264.7 was performed according to previous studies (Fu et al., 2019; Duan et al., 2021; Zhao et al., 2022). RAW 264.7 was seeded on 96-, 12-, or 6-well plates. LP pellets were collected after three centrifugal washed and incubated with the cells at a ratio of 10/1 macrophages or 100/1 macrophages for 1 h or 12 h. Polarization was determined by collecting cells or supernatants after washing thrice with PBS.

### Determination of cell viability

The cell-counting kit-8 (CCK-8) was used to determine cell viability (Liu et al., 2020). RAW 264.7 (5 × 10^4^ cells/well) were co-cultured in the absence or presence of LP (5 × 10^5^ or 5 × 10^6^ CFU/well) in 96-well plates. The culture medium was replaced with a 100 μl RPMI-1640 medium containing 10 μl of CCK-8 (Abcam, UK) and incubated at 37°C for 90 min. Finally, the optical density (OD) was measured at a wavelength of 450 nm, and cell viability was evaluated as (OD value of the stimulated cell-OD value of unstimulated cells)/(OD value of the unstimulated cell-OD value of blank cell) × 100 %.

### ELISA

Cell culture supernatant was collected for quantifying cytokines, such as TNF-α, IL-6, and IL-10, using an enzyme-linked immunosorbent assay (ELISA) according to the manufacturer's protocols for the Mouse ELISA kit (Meimian, China).

### Western blot

Cells were collected and lysed using radio immunoprecipitation assay (RIPA) lysis buffer (Beyotime Biotechnology). Samples were separated in a 12% SDS-PAGE and electro-transferred to polyvinylidene fluoride (PVDF) membranes (Thermo Scientific, USA). The membranes were blocked with 5% skim milk for 1 h and were incubated overnight at 4°C with primary antibodies against iNOS (1:1,000), Arg-1 (1:1,000), and β-actin (1:500) (Bioss, China). Following three washes with TBST, the membranes were incubated with secondary antibodies (1:20,000, ABP Biotech) for 2 h at room temperature. Immunogenicity testing was performed using an ECL kit (Sangon, China).

### Flow cytometry

Cells (~10^6^) were collected and labeled with CD86^+^-Pecy7 and CD206^+^-FITC antibodies (BD Biosciences). The stained cells were analyzed using a BD LSR-FORTESSA flow cytometer (BD Biosciences).

### Phagocytosis and bacterial killing assays

Phagocytosis and killing experiments were performed according to a previous protocol (Duan et al., 2021). Briefly, 5 × 10^5^ RAW 264.7 cells were co-cultured with LP (5 × 10^6^ CFU/well) for 1 h; cells were then infected with STM (5 × 10^6^ CFU/well) for 1 h at 37°C. Extracellular bacteria were isolated using RPMI-1640 medium containing gentamicin (50 μg/ml) for 1 h. To ensure the bactericidal efficiency induced by LP against STM, the medium was incubated for 1, 2, 4, and 6 h. After that, the cells were lysed in 1% Triton X-100 at the indicated time points. The intracellular STM were cultured on *Salmonella*–*Shigella* (SS) enumeration agar plates for 12 h at 37°C. To visualize the STM in cells, STM was stained with carboxyfluorescein succinimidyl ester (CFSE, 2.5 μM) and subsequently used to infect RAW 264.7. STM-CFSE was visualized using a fluorescence microscope. The cell nuclei were stained with 4, 6-diamidino-2-phenylindole (DAPI, 5 μg/ml) for 15 min, and 100 cells per field were counted. The phagocytic index was calculated as the average number of bacteria per cell.

### Bacterial interaction with bone marrow macrophages

Bone marrow macrophage (BMM) were routinely isolated and differentiated for 1 week in RPMI-1640 complete medium supplemented with 10 ng/ml M-CSF (PeproTech, USA; Fu et al., 2019). Briefly, BMMs were harvested from a 12-well plate (~10^5^ cells/well) as M0. M0 were cultured for 12 h in a medium containing IFN-γ (10 ng/ml) and LPS (10 ng/ml) to differentiate M0 into an M1-positive phenotype. BMM with M0 or M1 phenotypes were treated with LP (~10^6^ CFU/well) for 6 h and examined for markers associated with M1 polarization, such as CD86^+^, TNF-α, IL-6, IL-10, or bactericidal activity. The NF-κB pathway was evaluated through western blotting using primary antibodies against IκBα (1:2,000), *p*-IκBα (1:1,000), p65 (1:1,000), and *p*-p65 (1:1,000) (Bioss, China), as described previously. In addition, BMMs were cultured in a medium containing pyrrolidine dithiocarbamate ammonium (PDTC, 20 μM) for 1 h to inhibit NF-κB. The cells were stimulated with an M1 inducer (IFN-γ and LPS) or LP and examined for markers associated with M1 polarization, such as CD86^+^, TNF-α, IL-6, and IL-10.

### Protective effect of LP in C57BL/6 mice against STM

C57BL/6 mice were randomly divided into four groups (15 mice/group): (1) LP, intragastric inoculation of ~10^9^ CFU/mouse LP for 7 days; (2) STM, intragastric administration of ~10^8^ CFU/mouse STM 4 h after water administration on day 8; (3) LP + STM, oral administration of LP for 7 days and STM infection on day 8; and (4) control, LP was replaced with the same amount of PBS. Body weights and survival rates were monitored every day in 10 mice from each group. The remaining five mice were euthanized 3 days post-infection (pi), and the liver, spleen, and mesenteric lymph nodes (MLNs) were collected. The liver and spleen were weighed and homogenized in PBS, and bacterial loads were determined. Briefly, tissue suspension was collected on an SS agar plate and incubated at 37°C for 12 h.

### FCM analysis of macrophage polarization in the spleen and MLNs

Spleen and MLNs single-cell suspensions were prepared and stained with antibodies against F4/80-PE, CD80-APC, CD206-FITC, and CD86-PE-cy7 (BD, USA). Flow cytometry was performed to determine the proportion of CD80^+^ F4/80^+^, CD86^+^ F4/80^+^, and CD206^+^ F4/80^+^ double-positive cells in the spleen and MLNs.

### Histopathology

Colon segments were aseptically collected (1 cm) and the contents were rinsed with PBS. Colonic segments were fixed in 4% paraformaldehyde to prepare paraffin sections; the sections were stained with hematoxylin and eosin. Histopathological damage was scored blindly by a veterinary pathologist according to the criteria shown in Table 1 (Sarichai et al., 2020).

**Table 1:** Histopathology score criteria (Sarichai et al., 2020).

**Score**	**Neutrophils infiltration**	**Mononuclear leukocyte infiltration**	**Submucosal edema**	**Epithelial damage**	**Exudate**
**0**	No changes/0–5	No changes/0–5	No changes	No damages	No changes
**1**	6–20	6–10	Detectable (<10 %)	Desquamation	Slight accumulation
**2**	21–60	11–20	Mild (10–20 %)	Mild erosion, mild loss of goblet cells	Mild accumulation
**3**	61–100	21–40	Moderate (21–40 %)	Marked erosion, moderate loss of goblet cells	Moderate accumulation
**4**	>100	>40	Marked (>40 %)	Ulceration and marked loss of goblet cells	Marked accumulation

### Determination of cytokine levels in duodenal mucosa

The duodenal segments were sonicated in PBS supplemented with PMSF (333.3 mg/ml) and centrifuged for supernatant collection. ELISA kits were used to detect cytokines (such as TNF-α, IL-6, IL-1β, and IL-10).

### Statistical analysis

All data are presented as mean ± standard deviation. Data of STM counts between two groups were analyzed using an unpaired t-test. Statistical significance was processed using a one-way analysis of variance (ANOVA) with Tukey. All pictures were generated using the GraphPad Prism 8.0.1 software. ^*^p < 0.05, ^**^p < 0.01, ^***^p < 0.001 were considered significant and ns indicates not significant.

## Result

### LP affected polarization of RAW264.7 macrophage

**Figure 1:**
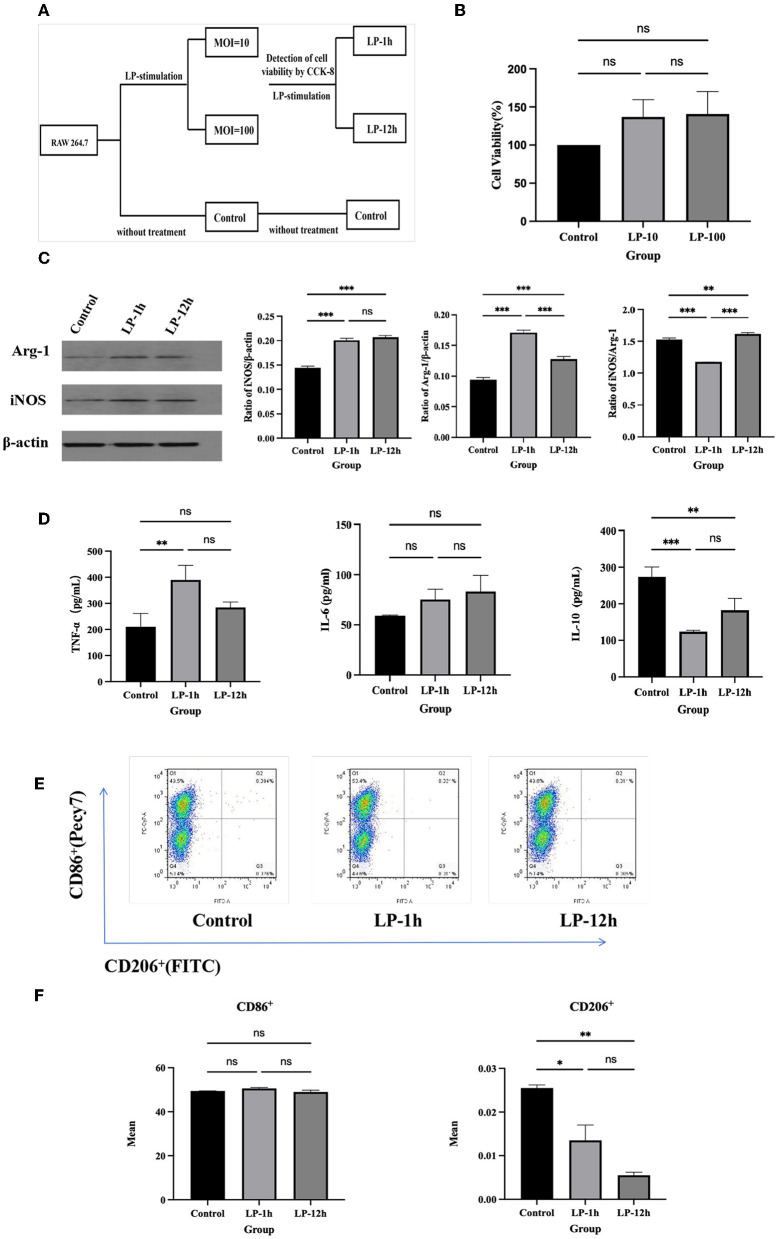
The effect of RAW 264.7 polarization induced by LP. RAW 264.7 (5 × 10^4^ cells/well) was inoculated into a 96-well plate and exposed to LP (5 × 10^5^ or 5 × 10^6^ CFU/well) at an MOI of 10 or 100 for 12 h. CCK-8 assay was performed to evaluate the safety of LP. RAW 264.7 (5 × 10^4^ cells) were then cultured with or without LP at an MOI of 10 for 1 h or 12 h, respectively, to observe the effect on macrophage polarization. **(A)** Diagram of cell grouping. **(B)** CCK-8 assay for cell viability. **(C)** Western blot was used to detect Arg-1 and iNOS protein expression in RAW 264.7 cells at 1 h or 12 h of LP stimulation. **(D)** Cytokine expression (including TNF-α, IL-6, and IL-10) in supernatants of LP-pretreated RAW 264.7 cells. **(E)** Representative analysis of the percentage of CD86^+^ or CD206^+^ cells in RAW 264.7 for each group, using FCM. **(F)** Statistical analysis of the proportion of CD86^+^ and CD206^+^ cells. Results are presented as means ± SD (data were analyzed using one-way ANOVA. ^ns^ not significant, *p < 0.05, **p < 0.01, ***p < 0.001).

To evaluate the safety of LP, RAW 264.7 (5 × 10^4^ cells/well) were co-cultured in the absence or presence of LP (5 × 10^5^ or 5 × 10^6^ CFU/well) for 12 h (Figure 1A). The CCK-8 assay results showed that there was no significant difference in viability between the RAW 264.7 normal group and the LP-exposed group (Figure 1B). Additionally, to investigate the effect of LP on macrophage polarization, we pre-treated RAW 264.7 with LP for 1 h or 12 h. Interestingly, both iNOS and Arg-1 were enhanced by LP stimulation for 1 h or 12 h, compared with that in the control. However, iNOS/Arg-1 levels were significantly elevated only after 12 h of LP stimulation (*p* < 0.001) (Figure 1C). One hour of LP stimulation promoted the secretion of TNF-α (*p* < 0.01) and IL-6 (*ns*) and significantly inhibited the production of IL-10 (*p* < 0.001) compared to that in the control group; the increase at 1 h was similar to that at 12 h (Figure 1D). RAW 264.7 stimulated with LP for 1 h showed a slight increase in the proportion of CD86^+^ compared to that in the control (ns). The percentage of CD206^+^ decreased in RAW 264.7 when stimulated with LP for 1 h (*p* < 0.05) and 12 h (*p* < 0.01) (Figures 1E,F). These data suggest that LP may affect the polarization of macrophages.

### LP promotes the phagocytic and bactericidal activity of RAW 264.7 against STM

**Figure 2:**
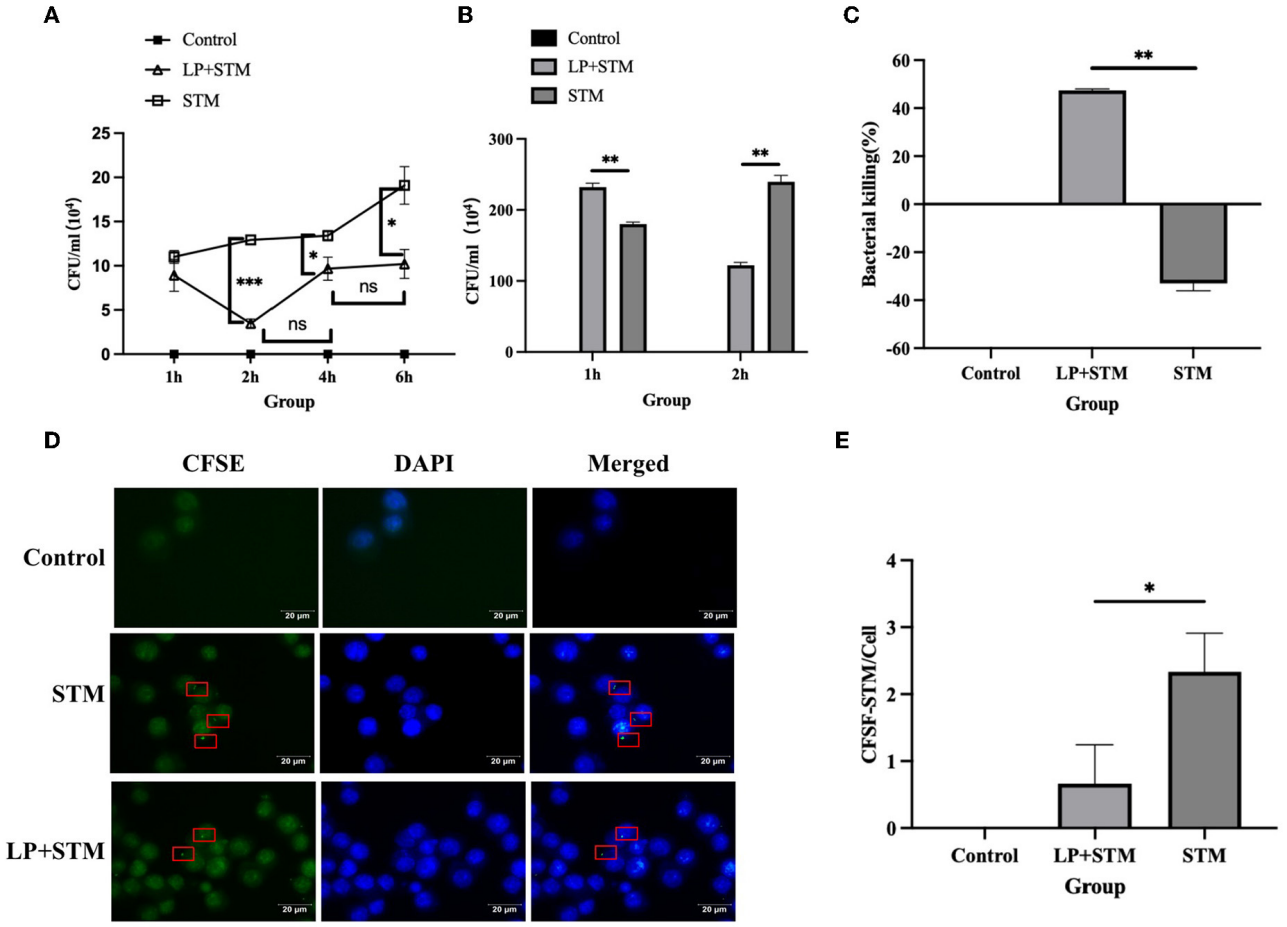
LP enhanced the ability of RAW264.7 to phagocytosis and kill STM. **(A)** Intracellular STM counts in RAW 264.7. RAW 264.7 (5 × 10^5^ cells/well) were pre-treated with LP (5 × 10^6^ CFU/well) or PBS for 1 h and then infected by STM (5 × 10^6^ CFU/well) for 1 h. After killing extracellular bacteria with gentamicin (50 μg/ml) RPMI-1640 medium for 1 h, cells incubated for 1, 2, 4, and 6 h were lysed onto an SS agar plate. **(B)** The number of intracellular STM at 1 h and 2 h pi of STM infection. **(C)** Histogram of bactericidal activity. Bactericidal activity is calculated according to the formula: bactericidal index = (number of bacteria incubated at 1 h – incubated at 2 h)/(number of bacteria incubated at 1 h) × 100 %. **(D)** CFSE-labeled STM in RAW 264.7 cells. RAW264.7 was incubated with LP for 1 h as described above. These cells were infected with STM and stained with cfse (STM-cfse) for an additional 1 h and DAPI for the nucleus for 15 min. **(E)** Histogram of the number of STM-cfse in each RAW 264.7 cell. Results are presented as means ± SD (data were analyzed using unpaired t test. *p < 0.05, **p < 0.01, ***p < 0.001).

The results showed that LP decreased the number of intracellular STM at 1 h, 2 h (*p* < 0.001), 4 h (*p* < 0.05), and 6 h pi (*p* < 0.05) of STM infection, especially at 2 h (Figure 2A). To calculate the phagocytosis index of cells, the number of intracellular STM at 1 h and 2 h were measured, and the result showed that LP significantly decreased the number of STM at 2 h pi of STM infection (*p* < 0.01) (Figure 2B). The bactericidal index of cells stimulated with LP was as high as 47.42%, while the cells without LP treatment were negative (Figure 2C). The phagocytosis assay showed CFSE-labeled STM in macrophages (Figure 2D). Phagocytic index analysis indicated that phagocytosis in the LP-pretreated (LP + STM) cells was less than that in the untreated (STM alone) cells at 2 h after the STM challenge (*p* < 0.05) (Figure 2E). These results collectively suggest that LP enhances the bactericidal activity of macrophages against STM infection.

### LP synergistically enhances M1 differentiation of BMMs

**Figure 3:**
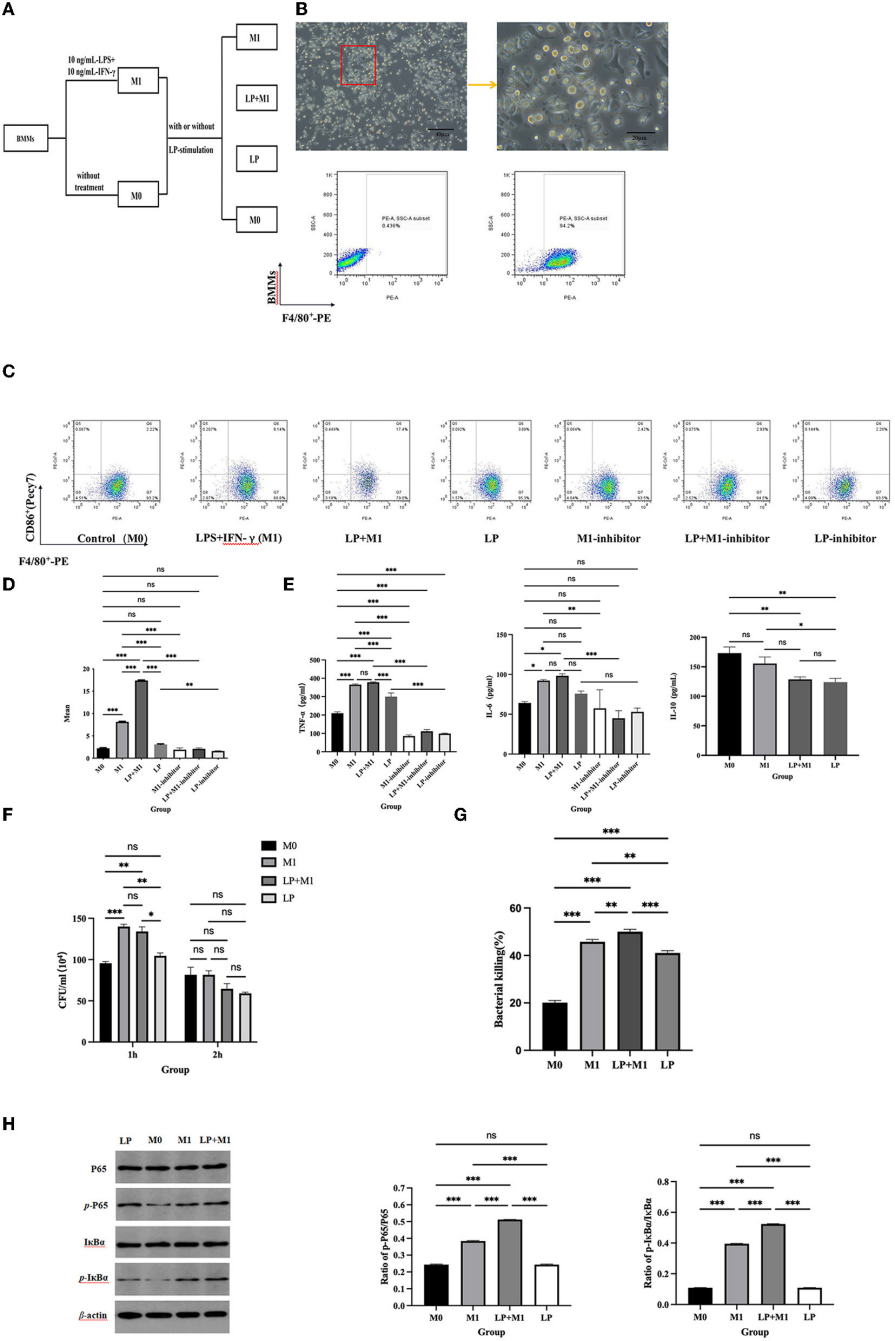
LP synergistically promoted M1 macrophage polarization and BMMs function. BMMs (M0) were isolated from mouse bone marrow and induced to culture by 10 ng/ml M-CSF for 7 days. BMMs were stimulated with 10 ng/ml IFN-γ and 10 ng/ml LPS (M1 inducer) for 12 h. M0 and M1 cells (~10^5^ cells/well) were exposed to LP (~10^6^ CFU/well) for 6 h, respectively. **(A)** Diagram of cell grouping. **(B)** Morphological characteristics of macrophages under a microscope, and percentage of F4/80^+^ cells using flow cytometry. **(C)** Representation of F4/80^+^CD86^+^ cells in BMMs from each group, using FCM. **(D)** Statistical analysis of F4/80^+^CD86^+^ percentage. **(E)** LP stimulated the expression of cytokines (including TNF-α, IL-6, and IL-10) in M0 and M1 supernatants. **(F)** STM counts in BMMs. M0 and M1 macrophages (5 × 10^5^/well) were pre-treated with LP (5 × 10^6^ CFU/well) or PBS for 6 h and then treated with STM (5 × 10^6^ CFU/well) or PBS for 1 h. After incubation for 1 h or 2 h, these cells were lysed into SS agar plates. **(G)** Histogram of bactericidal activity. Bactericidal activity is calculated according to the formula: Bactericidal index = (number of bacterial incubated at 1 h – number of bacterial incubated at 2 h)/number of bacterial incubated at 1h × 100 %. **(H)** Western blot was used to detect NF-κB protein synthesis in M0, M1, LP, and LP+M1 groups. Histogram of *p*-IκBα vs. *p*-p65 protein synthesis levels. Results are presented as means ± SD (data were analyzed using one-way ANOVA. ^ns^ not significant, *p < 0.05, **p < 0.01, ***p < 0.001).

After 7-days of differentiation, the adherent cells presented irregular spindle-like and prosthetic extensions, and the proportion of F4/80^+^ cells detected using FCM was 94.3%, indicating the successful induction of BMMs (Figure 3B). To investigate whether LP had a synergistic effect on M1 polarization in BMMs, M0 or M1 phenotypes of BMMs (~10^5^ cells/well) were stimulated with or without LP (~10^6^ CFU/well) for 6 h to detect M1 phenotype-related markers (Figure 3A). F4/80^+^CD86^+^ was significantly increased in the M1, LP, and LP+M1 groups compared with that in the M0 group (*p* < 0.01). Interestingly, the proportion of F4/80^+^CD86^+^ cells was significantly higher in the LP+M1 group compared to that in the M1 group (*p* < 0.001) (Figures 3C,D). In addition, the production of TNF-α or IL-6 increased in the LP group (TNF-α: *p* < 0.001, IL-6: ns), M1 group (TNF-α: *p* < 0.001, IL-6: *p* < 0.05), and LP+M1 group (TNF-α: *p* < 0.001, IL-6: *p* < 0.05) compared to that in the M0 group (*p* < 0.01). IL-10 secretion was reduced in the M1 (ns) and LP+M1 groups (*p* < 0.01). The levels of TNF-α and IL-6 in the LP+M1 treatment group were slightly increased than that in the M1-treatment group (ns). However, the levels of IL-10 were similar between the LP (*p* < 0.01) and LP+M1 (ns) treatments and lower than that in the M1 group (Figure 3E). The combination of LP and M1 promoted the bactericidal activity of macrophages against STM compared to that with M1 alone (Figure 3F). This suggests that LP itself does not affect M1 polarization as much as M1 inducers (IFN-γ and LPS) do, but LP synergistically enhances M1 polarization.

To determine whether LP-induced M1 polarization is mediated by the NF-κB signaling pathway, NF-κB activation was evaluated, followed by treatment with an inhibitor to stimulate M1 inducers (IFN-γ and LPS). We observed an increase in *p*-IκBα and *p*-p65 after 6 h of co-culture of BMMs (M0 or M1 phenotype) with LP (Figure 3H). In particular, the levels of *p*-IκBα and *p*-p65 were higher in the LP+M1 group compared to that in the M0 group (*p* < 0.001) or M1 group (*p* < 0.001) (Figure 3). However, these proteins were slightly increased in the LP group compared to that in M0 (ns) (Figure 3H). Interestingly, after supplementation with the NF-κB inhibitor, F4/80^+^CD86^+^, TNF-α, and IL-6 significantly decreased in the M1, LP, and LP+M1 groups compared to that in the group without inhibitor (*p* < 0.001) (Figure 3E). These results collectively demonstrate that LP induced M1 polarization through the NF-κB signaling pathway.

### LP promotes the protective effect on C578L/6 mice against STM

**Figure 4:**
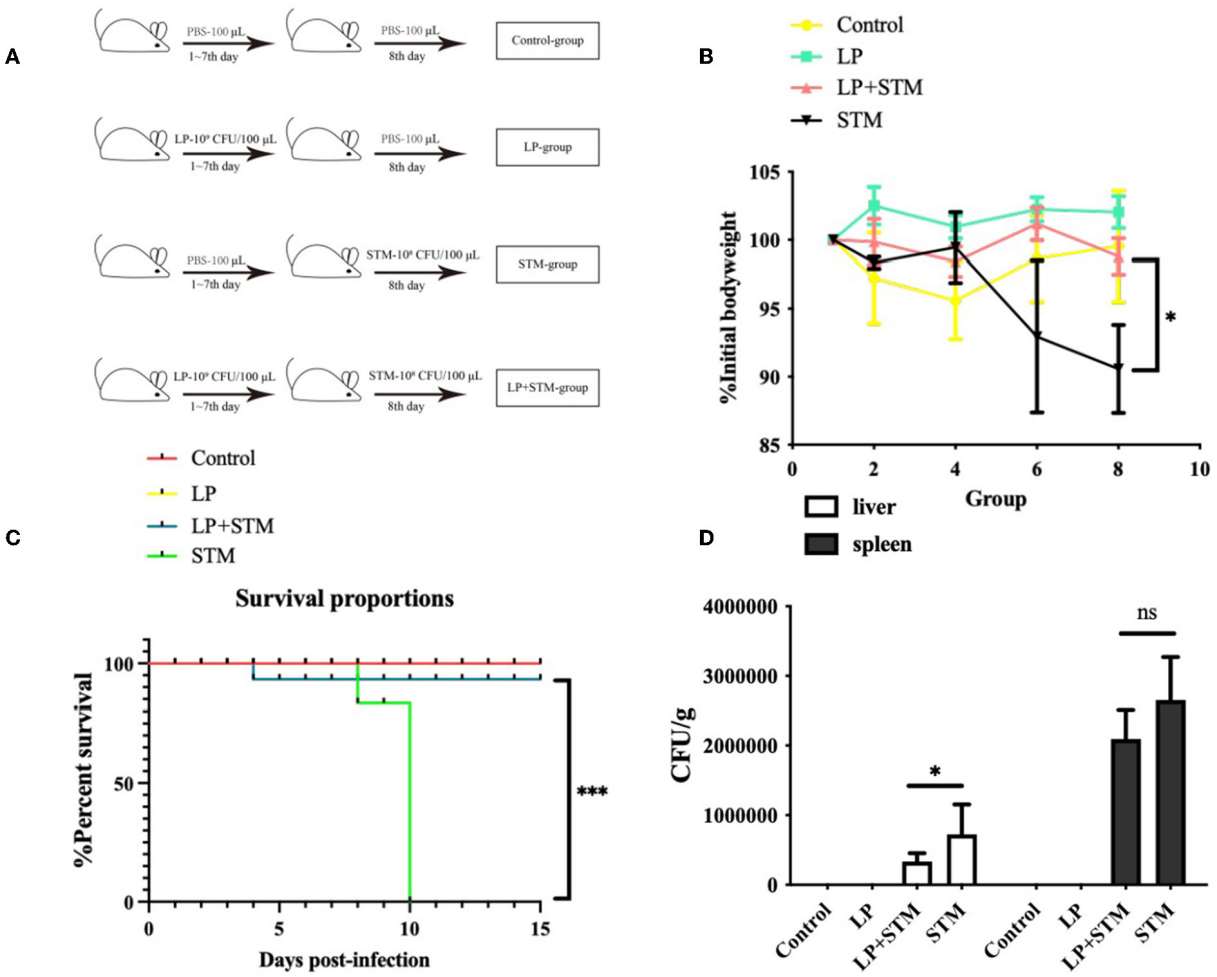
*Lactobacillus plantarum* pre-administration enhanced the protective effect *in vivo* of mice against STM. C57BL/6 mice were divided into four groups: LP, STM, LP + STM, and Control. LP, LP (~10^9^ CFU/100 μl) was intragastrically administered for 7 days; STM, STM (~10^8^ CFU/100 μl) was orally administered on day 8; LP + STM, mice were orally infected with LP for 7 days and infected with STM; for the Control, LP was replaced with PBS. **(A)** Mice grouping diagram. **(B)** Identification of body weight loss after infection for over 10 days with STM. **(C)** Mice survival rate was recorded 15 days after being infected with STM. **(D)** STM loads in spleen and liver. No STM colonies were observed in the liver or spleen of mice in the LP and Control groups. Results are presented as means ± SD (data were analyzed using survival analysis and unpaired t-test. ^ns^ not significant, *p < 0.05, **p < 0.01, ***p < 0.001).

Mice were intragastrically administered LP or PBS for 1 week, and an STM challenge was administered the following day. The results of the *in vivo* therapeutic trials in mice are shown in Figure 4A. Mice in the LP group experienced less weight loss and were similar to mice in the control group. The LP + STM group showed a decreasing trend before 4 dpi and a gradual increase thereafter. Mice in the STM group showed a significant decrease in body weight at 6 dpi (*p* < 0.05) compared to mice in the LP + STM group (Figure 4B). All mice in the STM group died; the survival rate of mice in the LP + STM group was as high as 90%, which lasted until 15 dpi. No mice died during this study in the LP and control groups (Figure 4C). Bacterial loads in the liver or spleen of mice at 3 dpi are shown in Figure 4D. Compared to the STM group, the LP + STM group showed a decreased STM burden in the spleen (ns) and liver (*p* < 0.05).

### LP promotes M1 polarization of macrophages in the spleen and MLNs

**Figure 5:**
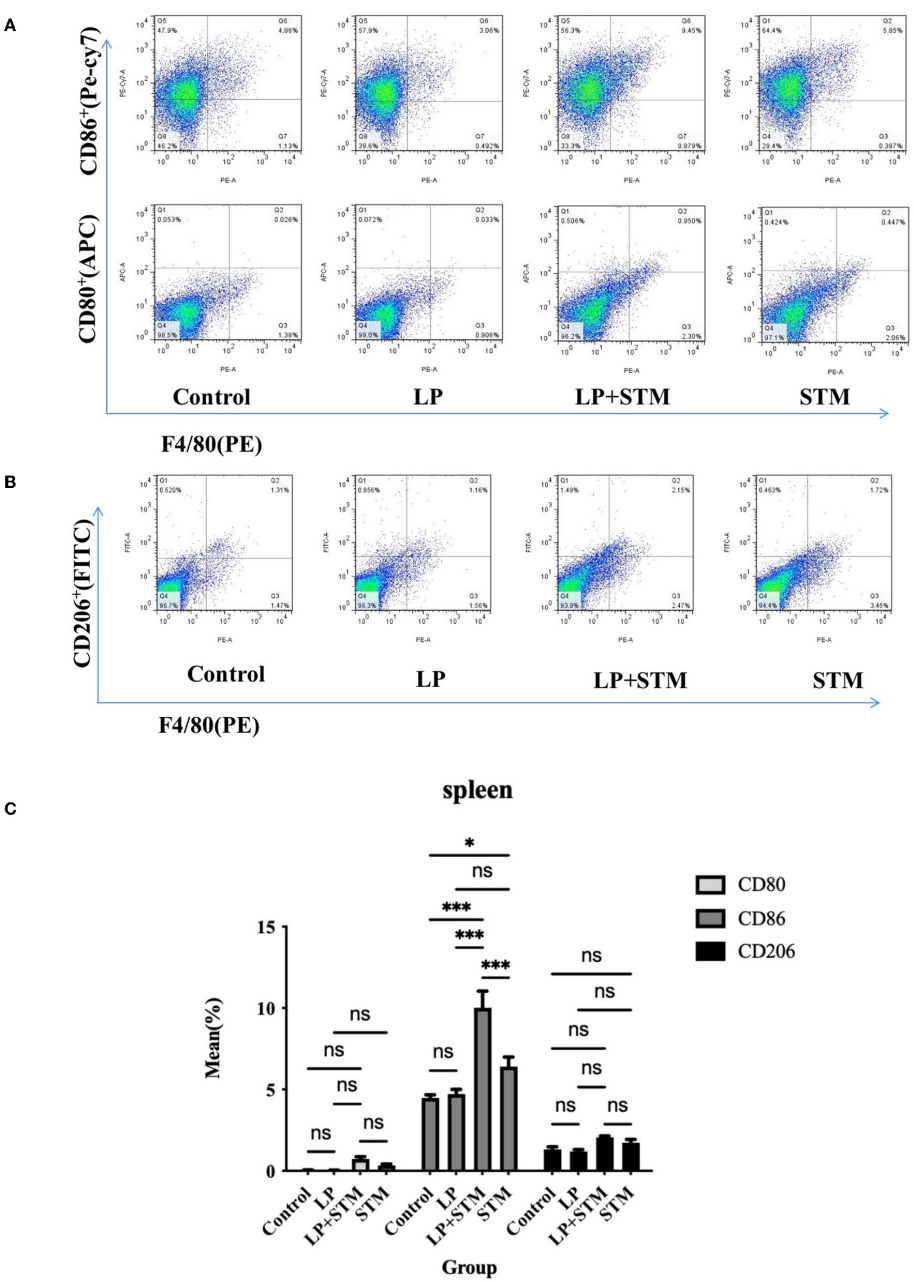
LP pretreatment promoted M1 polarization in mouse spleen macrophages. **(A,B)** Representative analysis of percentage of F4/80^+^ CD80^+^ or F4/80^+^ CD86^+^ or F4/80^+^ CD206^+^ cells detected by flow cytometry in spleens from each group. **(C)** Statistics of the proportion of F4/80^+^ CD80^+^, F4/80^+^ ${\text{CD}86}_{,}^{+}$ and F4/80^+^ CD206^+^ cells. Results are presented as means ± SD (data were analyzed using one-way ANOVA. ^ns^ not significant, *p < 0.05, **p < 0.01, ***p < 0.001).

**Figure 6:**
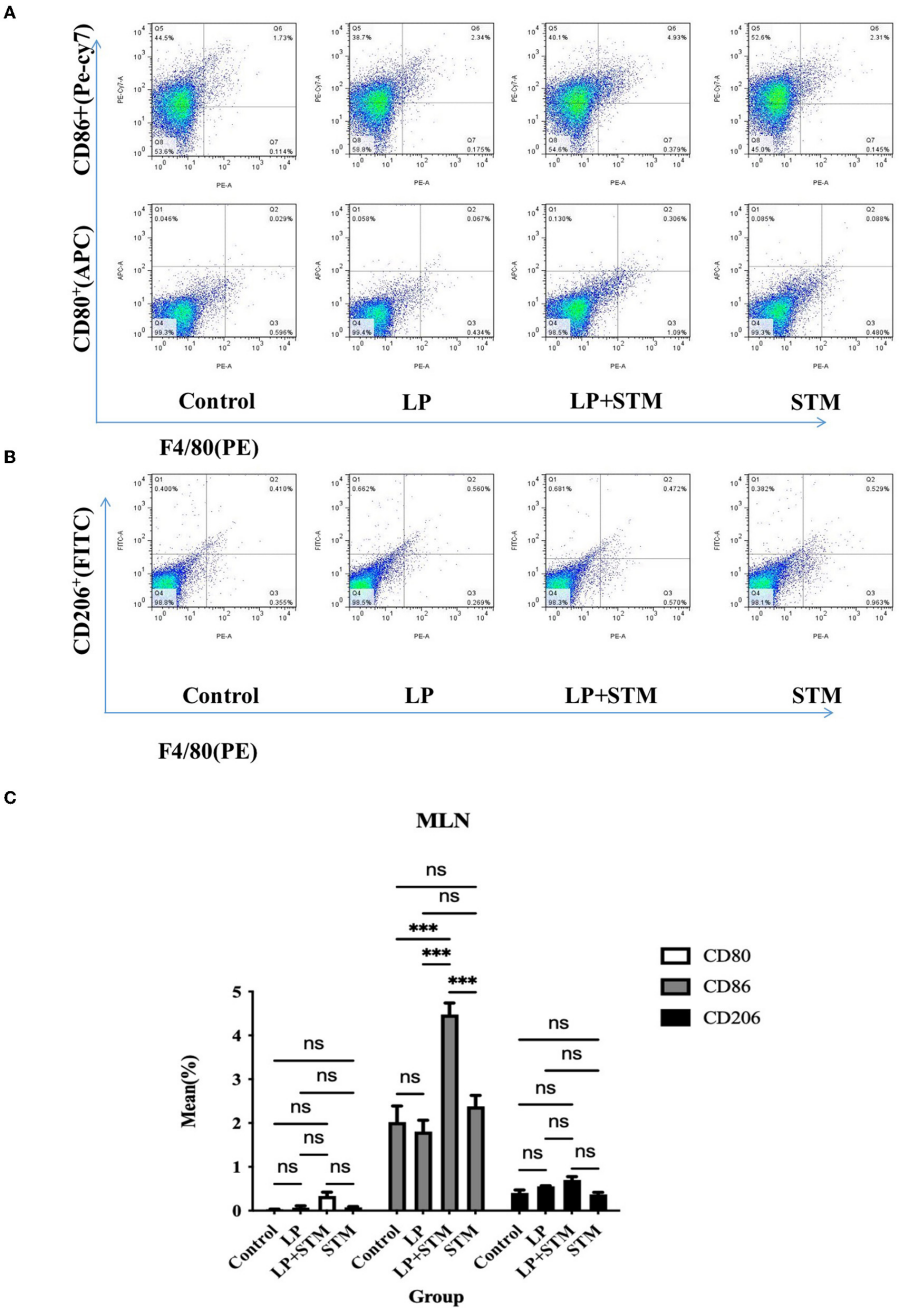
LP pretreatment promoted M1 polarization in mouse MLN macrophages. **(A,B)** FCM analysis of the percentage of F4/80^+^ CD80^+^ or F4/80^+^ CD86^+^ or F4/80^+^ CD206^+^ cells in MLN from each group. **(C)** Statistics of proportion of F4/80^+^ CD80^+^, F4/80^+^ CD86^+^, F4/80^+^ CD206^+^ cells. Results are presented as means ± SD (data were analyzed using one-way ANOVA. ^ns^ not significant, *p < 0.05, **p < 0.01, ***p < 0.001).

To further clarify the effect of LP on macrophage polarization, the proportion of F4/80^+^CD80^+^, F4/80^+^CD86^+^, and F4/80^+^CD206^+^ cells in the mouse spleen and MLNs were analyzed using FCM at 3 dpi (Figures 5A,B, 6A,B).

The proportion of F4/80^+^CD86^+^ cells in the spleen of mice from the LP + STM group was higher than that in the control (*p* < 0.001), STM (*p* < 0.001), and LP groups (*p* < 0.001), respectively. LP group or STM group showed an increase compared with the control group (LP: ns; STM: *p* < 0.05, respectively). The percentage of F4/80^+^CD80^+^ and F4/80^+^CD206 in the spleen were similar among these groups (ns) (Figure 5C).

In MLNs, the proportion of F4/80^+^CD86^+^ cells from the LP + STM group was higher than that in the control (*p* < 0.001), STM (*p* < 0.001), and LP groups (*p* < 0.001), respectively. LP group or STM group only showed a slight increase compared with the control group (ns). The percentage of F4/80^+^CD80^+^ and F4/80^+^CD206 were similar among these groups (ns) (Figure 6C).

### LP alleviates intestinal inflammatory damage

**Figure 7:**
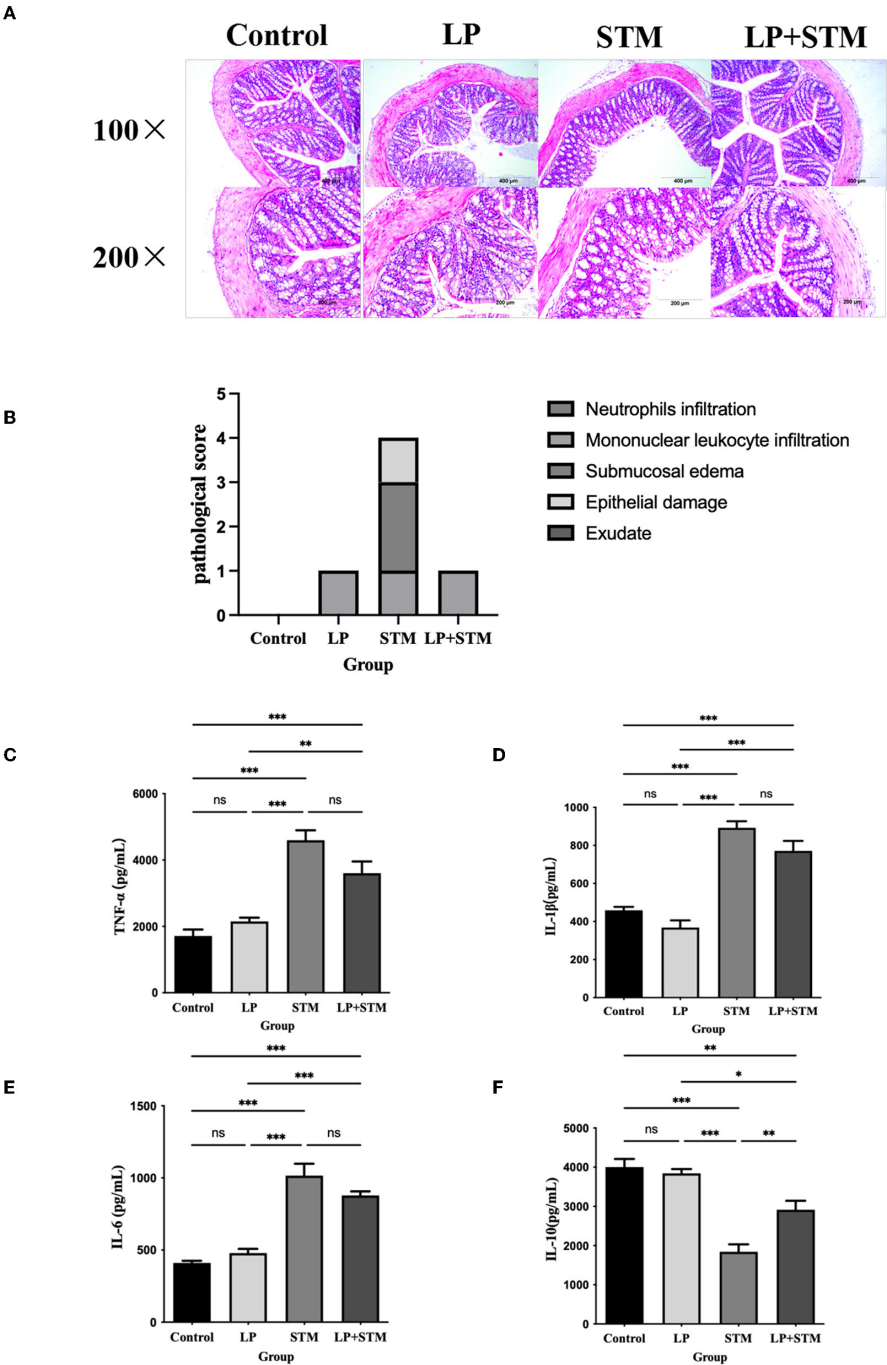
LP alleviated the intestinal inflammatory injury induced by STM in mice. **(A)** The mice colon was performed by H&E (100 × , 200 × ) staining, to detect the intestinal pathological changes in each group of mice after STM infection. Scale bar: 400 μm. **(B)** Histograms of pathological scores. **(C–F)** ELISA kits were used to detect the expression of cytokines (TNF-α, IL-6, IL-1β and IL-10) secreted by the intestinal mucosa of mice in the control, LP, LP + STM and STM groups. Results are presented as means ± SD (Data were analyzed using one-way ANOVA. ^ns^ not significant, *p < 0.05, **p < 0.01, ***p < 0.001).

To determine whether LP caused inflammatory damage in the resistance to STM infection, the histopathological scores of the mouse colon were evaluated, and cytokine levels were determined (Figure 7). The intestinal structure of mice in the LP group was similar to that of the mice in the control group. Mice infected with STM displayed a thinner intestinal wall, shedding of intestinal villi, and submucosal edema. LP pretreatment alleviated the intestinal damage caused by STM, as indicated by the intact structure of the glands and villi. Several inflammatory cell infiltrations were observed in the serous or mucosal layer of the LP group, a submucosal layer of the STM group, and the mucosal layer of the LP-STM group (Figure 7A). The histopathological scores of the LP + STM group were similar to the LP group, lower than STM group (Figure 7B).

The levels of the cytokines, IL-1β, IL-6, and TNF-α increased, while that of IL-10 decreased in both the STM and LP + STM groups compared to that in the control group (*p* < 0.001). However, LP alone did not affect the production of these cytokines, which was similar to that in the control. LP + STM treatment notably increased IL-10 secretion, compared to that in the STM group (*p* < 0.01), and slightly decreased the secretion of pro-inflammatory cytokines (such as IL-1β, IL-6, and TNF-α) (Figures 7C–F).

## Discussion

STM is an intracellular pathogen that can cause gastroenteritis and systemic infection in humans and animals. It can invade a variety of host cells, such as intestinal epithelial cells, macrophages, or dendritic cells for replicative culture, especially macrophages (Fàbrega and Vila, 2013; Rocha-Ramírez et al., 2017). After STM penetrates the intestinal epithelial barrier, phagocytic cells infect the lamina propria and then enter macrophages to diffuse to the liver and spleen, causing systemic infection (Gogoi et al., 2019). The intracellular infection ability helps STM evade host immune defense. In this study, LP itself or that combined with BMMs displayed bactericidal activity against STM *in vitro*. *In vivo*, LP protected C57BL/6 mice against STM. Probiotics can initiate immune defense and exert immunomodulatory effects by regulating cytokine production and driving macrophage polarization (Kang and Im, 2015; Yang et al., 2017; Theret et al., 2019). Bacterial co-culture experiments were performed on the macrophages to investigate such interactions. In this study, even when RAW 264.7 cells were co-cultured with LP at 100/1 bacteria/cell for 12 h, the cell viability remained intact and the CCK-8 assay results were similar to that of normal cells.

Specific probiotic species may have different polarization effects. In the case of *Lactobacillus plantarum*, some strains (such as KFCC11389P, HY7712, and CAU1055) are considered anti-inflammatory mediators (Chon et al., 2009; Jang et al., 2013; Choi et al., 2019). However, some others (such as NRRL B-4496, RS20D, and Ln1) exhibit antimicrobial activity by promoting pro-inflammatory cytokines or signaling pathways (NF-κB or MAPK), but are non-toxic (Zhu et al., 2019; Arrioja-Bretón et al., 2020; Jang et al., 2020). Even when macrophages are exposed to the same probiotic strain, different results may be obtained, depending on the macrophage status (Habil et al., 2011).

In this study, we observed different effects on RAW 264.7 polarization in LP stimulation for 1 h or 12 h. LP stimulation increased both iNOS and Arg-1. Arg-1 and iNOS compete to convert l-arginine. Therefore, iNOS/Arg-1 were probably associated with M1 macrophage polarization (Brigo et al., 2021). This study showed an increase in iNOS/Arg-1 levels for 12 h-stimulation, but a decrease for 1 h-stimulation. LP increased the secretion of TNF-α or IL-6 to a different extent, and notably decreased the expression of IL-10 and CD206. Therefore, LP itself might slightly modulate M1 macrophage polarization, but it is uncertain. These results are partly consistent with those of previous studies on LGG, *Lactobacillus plantarum* RS-09, and *Lactobacillus johnsonii* polarizing M1 macrophages against pathogen infection (Affar et al., 2018; Wang et al., 2020; Zhao et al., 2022). LGG triggers M1 polarization and induces both pro-inflammatory and anti-inflammatory cytokines in BMM, which aid in maintaining immune balance and homeostasis (Wang et al., 2020).

To make sure the exact effect of LP on macrophage polarization, BMMs were induced to the M1-positive phenotype with LPS and IFN-γ (M1 positive control). At the same time, a synergistic effect was also investigated. In this study, LP also did not display an obvious modulatory effect on M1 polarization compared with the M1-positive macrophage. Interestingly, LP co-cultured with the M1-positive phenotype presented a higher degree of M1 polarization than LP or M1 alone, suggesting a synergistic effect.

Among the various signaling pathways involved in macrophage differentiation, nuclear factor NF-κB (NF-κB) signaling plays an important role in the regulation of M1 polarization and inflammatory responses (Luo et al., 2020). Some components of probiotic strains (peptidoglycan, lipoteichoic acids, and cell wall) are recognized by TLRs, thereby triggering NF-κB (Takeda and Akira, 2004). The latter induces the secretion of inflammatory cytokines (such as TNF-α, IL-1β, and iNOS), which are biomarkers of M1 macrophages (Cunha et al., 2016). In this study, LP or LP in combination with M1 (LP+M1) triggered the NF-κB signaling pathway, as indicated by the increase in *p*-IκBα and *p*-p65. In addition, NF-κB inhibitors prevented LP from inducing the biomarkers of M1 macrophages, as indicated by the decreased levels of CD86^+^, TNF-α, and IL-6. Therefore, LP cooperatively polarizes the M1 phenotype through NF-κB.

In addition, M1 macrophage polarization is associated with inflammatory responses. Severe inflammation can induce host damage or cytokine storms (Kwon et al., 2020). Therefore, it is necessary to confirm whether LP causes excessive inflammatory damage. We assessed the protective effect of *Lactobacillus plantarum* against STM infection in C57BL/6 mice. We observed a protective effect of LP, as indicated by decreased weight loss and mortality and reduced STM burden in the liver. We observed dual regulation of mouse spleen and MLNs macrophages by LP, including M1 and M2 macrophage polarization, using FCM analysis. Overall, LP promoted M1 polarization, which is consistent with the results from earlier studies. *Bacillus amyloliquefaciens* induces M1 and M2 macrophage polarization in the cecum, thereby inhibiting *Salmonella* infection (Fu et al., 2019). Based on intestinal histopathology, LP treatment did not induce intestinal inflammatory damage in mice and did not significantly promote the production of inflammatory cytokines. Compared to STM treatment alone, LP + STM treatment significantly increased IL-10 secretion and slightly decreased inflammatory cytokine secretion. Therefore, LP exhibits a protective effect in limiting STM infection, without an inflammatory insult. Macrophage polarization is essential for regulating both innate and adaptive immune responses (Sica and Mantovani, 2012). LP may be involved in regulating macrophage polarization and in mediating adaptive immunity, such as activation of Th1, Th2, and Th17 cells; these should be elucidated further in future studies. In this study, we focused on the interaction between macrophages and LP. In summary, LP is promising to prevent and treat intestinal infections caused by pathogenic bacteria such as *Salmonella*.

## Conclusion

LP enhanced resistance against *S*. Typhimurium in C57BL/6 mice without causing inflammatory damage. These findings will lay the foundation for developing prevention and treatment strategies against *S*. Typhimurium infections in the future.

## Data availability statement

The original contributions presented in the study are included in the article/supplementary material, further inquiries can be directed to the corresponding author.

## Ethics statement

The animal study was reviewed and approved by the Animal Welfare and Research Ethics Committee of Jilin Agricultural University.

## Author contributions

JH and CW designed the experiment. BD and RL completed the experiments and wrote the paper. YS, AS, and ZT analyzed the data. All authors reviewed the results and approved the manuscript.

## Funding

This study was funded by the Jilin Province Science and Technology Project (20200201109JC and YDZJ202102CXJD029), the China Agriculture Research System of MOF and MARA (CARS-35), and the National Natural Science Foundation of China (U21A20261).

## Conflict of interest

The authors declare that the research was conducted in the absence of any commercial or financial relationships that could be construed as a potential conflict of interest.

The Reviewer JQ declared a shared affiliation with the authors at the time of the review.

## Publisher's note

All claims expressed in this article are solely those of the authors and do not necessarily represent those of their affiliated organizations, or those of the publisher, the editors and the reviewers. Any product that may be evaluated in this article, or claim that may be made by its manufacturer, is not guaranteed or endorsed by the publisher.
